# The possible role of methylglyoxal metabolism in cancer

**DOI:** 10.1080/14756366.2021.1972994

**Published:** 2021-09-13

**Authors:** Khalid O. Alfarouk, Saad S. Alqahtani, Saeed Alshahrani, Jakob Morgenstern, Claudiu T. Supuran, Stephan J. Reshkin

**Affiliations:** a Department of Evolutionary Pharmacology, and Tumor Metabolism, Hala Alfarouk Cancer Center, Khartoum, Sudan; b Pharmacy Practice Research Unit, Clinical Pharmacy Department, College of Pharmacy, Jazan University, Jazan, KSA; c Pharmacology and Toxicology Department, College of Pharmacy, Jazan University, Jazan, KSA; d Department of Internal Medicine I, Endocrinology and Metabolism, Heidelberg University, Germany; e Neurofarba Department, Universita Degli Studi di Firenze, Florence, Italy; f Department of Bioscience, Biotechnology and Biopharmaceutics, University of Bari, Bari, Italy

**Keywords:** Methylglyoxal, cancer, enzyme, pH, redox

## Abstract

Tumours reprogram their metabolism to acquire an evolutionary advantage over normal cells. However, not all such metabolic pathways support energy production. An example of these metabolic pathways is the Methylglyoxal (MG) one. This pathway helps maintain the redox state, and it might act as a phosphate sensor that monitors the intracellular phosphate levels. In this work, we discuss the biochemical step of the MG pathway and interrelate it with cancer.

## Introduction

Reactive oxygen species (ROS) are highly reactive chemical species that target various biomolecules within the cell. ROS examples include superoxides, peroxides, singlet oxygen, hydroxyl radical, alpha-oxygen, and alkoxyl radicals^1–3^. The prevailing unifying scientific theory is that ROS, especially at lower levels, supports malignant transformation, carcinogenesis, and invasion, which supports metastatic transformation^4–8^. However, ROS at a higher dosage inhibits tumour growth, with some anticancer agents' primary mode of action being ROS-induced cell injury^8^^,^^9^. Such a paradox and biphasic or dual role depending on the dose is termed “hormesis”^10^. The intracellular NADPH level is one of the key determinants that manipulates the ROS hormesis. Besides the pentose phosphate pathway (PPP), the MG pathway is an additional pathway that contributes to NADPH pooling of the cells.

Many tumour cells rely on anaerobic glycolysis even in presence of oxygen, an effect which is called “Warburg metabolism”. The methylglyoxal pathway (MG) is branching from the glycolysis pathway to manage the redox state of the cell rather than contributing to the production of energy in the form of ATP.

**Figure 1.**
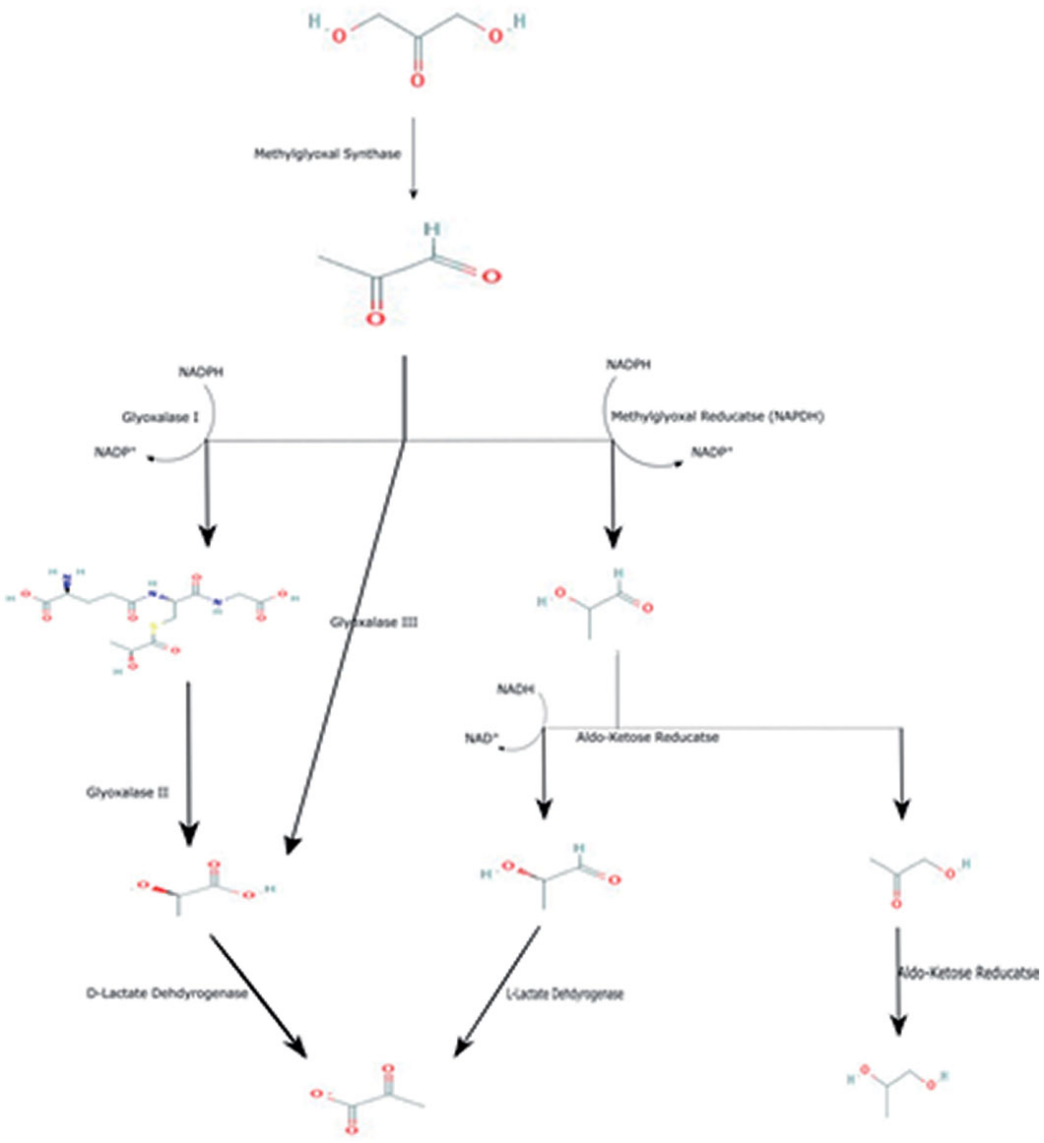
Summarises the biochemical pathway of methylglyoxal metabolism.

The MG pathway occurs in a series of steps that regulate the intracellular NADPH content, and it can also act as a phosphate sensor. MG is metabolised mainly either glutathione-dependent or glutathione-independent pathway, as follow (See Figure 1).

## Branching of glycolysis

Glycolysis is composed of two parts: the first one is the preparatory phase, followed by the second part, called the pay-off phase^11^. The pay-off part starts with forming D-glyceraldehyde 3-phosphate, which it is a crossroad of many biochemical pathways, including glycolysis^11^, pentose phosphate pathway^12^, as well as methylglyoxal metabolic pathway, as well as photosynthesis^13^^,^^14^.

D-glyceraldehyde 3-phosphate is isomerised to dihydroxyacetone phosphate (DHAP) by triosephosphate isomerase^11^. After that, DHAP is converted to MG (2-oxoaldehyde) and phosphate by the methylglyoxal synthase enzyme (MGS) activity.

MGS is also known as glycerone-phosphate phosphate-lyase (methylglyoxal-forming). Although MGS is a bacterial enzyme, early data showed that MGS was isolated from the goat liver^15^.

The optimum pH for MGS activity is 7.5, *i.e.,* alkaline pH^16^^,^^17^.

Phosphate acts as a competitive allosteric inhibitor of MGS. Some data concludes that the methylglyoxal pathway supports cells by phosphate and acts as a phosphate sensor^18^^,^^19^. ATP, 3-phosphoglycerate, and phosphoenolpyruvate inhibit MGS^15^^,^^16^. Therefore, it can be concluded that the MG pathway does not co-occur with the pay-off phase of the glycolysis pathway^11^. Other MGS inhibitors include: phosphoglycolohydroxamic acid^20^.

MG can be formed via several biochemical pathways^5^^,^^6^. MG is involved in many disorders including, cancer, diabetes, CNS disorders, etc.^21^. MG is a highly toxic compound^22–24^, and therefore, the body detoxifies the MG either through glutathione-dependent or glutathione-independent pathways.

### Glutathione-Dependent pathway

#### Lactoylglutathione:

MG is isomerised to hemithioacetal adducts and then form (R)-S-lactoylglutathione spontaneously in the presence of glutathione. The reaction is catalysed by a lactoylglutathione lyase (glyoxalase I)^25–28^.

The optimum pH for Glyoxalase I (GLO1) is broad, but generally, the optimum pH is alkaline around 8^29^.

GLO1 is over-expressed in many cancer types, such as, lung, colon, prostate, etc.^30–32^ GLO1 is also involved in their growth and progression, and resistance to the treatment^33–37^. GLO1 inhibition showed promising results as anti-tumour property^21^, as well as re-sensitizes the resistant tumours to the treatment^38^.

GLO1 inhibitors include 4–(7-azaindole)-substituted 6-phenyl-N-hydroxypyridones, Flavonoids, S-bromobenzylglutathione cyclopentyl diester (BrBzGCp2), and Curcumin^21^^,^^39–42^. Other GLO1 inhibitors include Ionising radiation,^43^, and nitric oxide (NO)^44^.

One of the supported observations is that GLO-1 is highly associated with tumorigenesis and tumour invasion^45^, where GLO-1 is GSH dependent and NADPH-dependent methylglyoxal reductase does not utilise GSH (see below).

#### D-Lactate

(R)-S-lactoylglutathione in the presence of water produced reduced glutathione and D-lactate via Hydroxyacylglutathione hydrolase (glyoxalase II)^46^.

In cancer, the role of GLO2 might be more complex. Although, tumour suppressor genes, e.g., p63 and p73, up-regulate GLO2 expression by tumour genes, GLO2 supported pro-survival rate rather than apoptosis, which is paradoxical. Cytosolic GLO2, not mitochondrial, prevents the MG induced-apoptosis^47^. Further contradiction is coming where GLO2 expression is lower in cancerous tissues than the normal parent tissue that might delve into other mysteries^48^. Therefore, it will be wisely to reveal that GLO2 expression is associated with growth arrest. One of the suggested answers that release this chain sinnet knot is that the correlation between (i) D-lactate (presence of GLO2 supports D-lactate production), (ii) reduced glutathione (absence of GLO2 prevent the reduced GSH recycle), and (iii) the state of the cell (phases of cell cycle, whether in growth phase, or proliferation, or even dormancy), in a way that solves the redox paradox^31^^,^^49–53^. At the same time, the glutathione either supports the cell proliferation by diminishing the reactive oxygen species that initiate the programmed cell death or preventing the malignant transformation^12^^,^^54^^,^^55^.

Although the optimum pH for GLO2 is broad from 6.8–7.5^46^, it yet shifted towards alkalinity. Also, cytoplasmic acidification is accompanied by a subsequent decrease in its activity^56^.

S-carbobenzoxyglutathione is one of many GLO2 inhibitors^57^ (and for further information ref Al-Shar’i et al.^58^).

D-lactate is a toxic substance associated with many diseases, including short-bowel syndrome, D-Lactic acidosis, and neurotoxicity^59^^,^^60^. Potentially, D-Lactate might be metabolised to pyruvate via the putative human D-lactate dehydrogenase^61–63^, or excreted extracellularly^63–66^, or even recycled back to MG (MG-Shunt)^67–72^. Some form of probiotics, e.g., *lactobacillus sp*. has D-lactate dehydrogenase activity, which might utilise the D-lactate, and therefore benefits during D—Lactic acidosis^73^.

### Glutathione-independent pathway

Due to the activity of NADPH dependent Aldose-ketose Reductase (AKR), MG can be metabolised into:

#### Lactaldehyde formation

In the presence of NADPH, AKR converts MG to lactaldehyde and produces NADP^+^. The NADP^+^ might be re-cycled to its reduced form (NADPH) using the pentose phosphate pathway (PPP)^12^. Therefore, the possible crosstalk between the MG and PPP is likely in the cell's physiology to manage the cell's redox state. In other words, there is a possibility of MG-PPP shunt to restore the NADPH.

AKR (NADPH) is also called NADPH-dependent methylglyoxal reductase Gre2, lactaldehyde:NADP^+^ oxidoreductase, and lactaldehyde dehydrogenase (NADP^+^).

The optimum pH for AKR (NADPH) is 6.5^74^, and the range is 5 to 7.5^75^, which moves towards the acidic pH. Therefore, it would be wise to reveal if the AKR (NADPH) is associated with either (i) cellular arrest neurodegeneration and/or renal impairment in case of acidic pH_i_^76–79^ or (ii) cellular senescence in case of alkaline pH_i_, and so the latter support the possibility of malignant transformation too^80–83^.

NADP^+^ inhibits NADPH-dependent MG-reductase; therefore it’s a negative feedback mechanism^75^^,^^84^. Calcium ion and 2-mercaptoethanol are examples of NADPH reductase inhibitors^75^^,^^84^.

##### Formation of lactic acid

In the presence of NAD^+^, Lactaldehyde is converted to L-lactate by aldehyde dehydrogenase (ALDH) to produce – Lactate and NADH.

Aldehyde dehydrogenase is overexpressed in cancer^85^ and associated with resistance to chemotherapy and radiotherapy, as well^86^.

Dyclonine, N,N‑diethylaminobenzaldehyde is an example of an ALDH inhibitor^86^^,^^87^. The optimum pH is around 7.4^88^.

##### Acetol formation

MG is converted to hydroxyacetone (acetol) via Aldo–keto reductase (AKR)^89^. AKR summarizes a broad family of oxidoreductase enzymes with varying capacities for the detoxification of MG^36^.

The AKR metabolises the MG, and the product is 95% acetol and 5% D-lactaldehyde^90^. Acetol is further metabolised to L-1,2-propanediol^90^ by the same enzyme^90^.

The optimum pH for AKR depends on the organism, tissue within the organism, etc. that might reflect enzymatic resilience in its activity to confers the organismal adaptability (evolutionary advantageous), e.g., the optimum pH of AKR in *Helicobacter* is in a range from 4–9, the optimum one is 5.5^91^, however, in more complex organisms the optimum is more basic in the small intestine^92^. Therefore, it will be challenging to detect or estimate the exact pH of AKR in cancer cells as these are characterised by their heterogeneity^93^.

AKR is overexpressed in many types of cancer, such as lung, uterine, colorectal, etc.^92^.

For AKR inhibitors, the Pharmacodiagnostics approach should be implemented for the rational use of selection for example, for

- AKR1B1 is inhibited by epalrestat^94^
- AKR1C1 is inhibited by 3-bromo-5-phenylsalicylic acid^95^.
- AKR1C3 is inhibited by cinnamic acid^96^^,^^97^.

## Notes on the MG metabolic pathway

Based on the reaction-diffusion kinetics, tumour neoplasm could be seen as multiple habitats. Tumour neoplasms show at least cline evolution from the macro-blood vessel (tumour cord). Therefore, tumour cells reprogram their metabolic machineries due to glucose, oxygen diffusion, and the lack of efficient removal of the metabolites (adaptive evolution)^93^^,^^98^^,^^99^. Therefore, it will not be surprising if the multi-regional biopsy to diagnose the tumours will not find the expression of the enzymes that are involved in MG metabolic pathways to the same degree (see Table 1), which is entirely predictable in the MG metabolic pathway as MG has a negative effect on the vasculature^100^.

**Table 1.** Shows the different set of enzymes involved in methylglyoxal metabolic pathway.

Enzyme	Optimum pH	Possible inhibitor(s)
Methylglyoxal synthase	7.5^16^^,^^17^	Phosphoenolpyruvate inhibit MGS^15^^,^^16^, phosphoglycolohydroxamic acid^20^
Glyoxalase I	8^29^	-(7-azaindole)-substituted 6-phenyl-N-hydroxypyridones, Flavonoids, S-bromobenzylglutathione cyclopentyl diester (BrBzGCp2), and Curcumin^21^^,^^39–42^ Nitric oxide (NO) also inhibits GLO1^44^.
Glyoxalase II	6.8-7.5^46^	S-carbobenzoxyglutathione^57^
Methylglyoxal reductase (NADPH)	5 to 7.5^75^	NADP^+^, Ca^2+^ and 2-mercaptoethanol^75^^,^^84^
Aldehyde dehydrogenase	7.4^88^	Dyclonine, N,N-diethylaminobenzaldehyde^86^^,^^87^
Aldo–keto reductase (AKR)	ND*	Epalrestat inhibits AKR1B1^94^ 3-bromo-5-phenylsalicylic acid inhibits AKR1C1^95^ cinnamic acid inhibits AKR1C3^96^^,^^97^

ND*: Not Determined.

Also, due to the reaction-diffusion kinetics, the hypoxic, necrotic regions within the tumour due to accumulation of lactate, and decreasing oxygen supply -at farther area from the blood vessel- the production of ROS increases^101–103^, and this might result in increasing the activity of NADPH oxidase (primary cellular source of ROS production)^104–107^. Therefore, the stimulation of oxidative stress-reducing agents is initiating the NADPH oxidase – MG metabolic pathway cross-talk, which has risen to come in a way that might confer the cancer cell survival^12^^,^^108–112^.

## Concluding remarks and future perspectives

MG is an intermediate product of many cross-roads’ biochemical pathways. The methylglyoxal products are toxic and must be detoxified consequently into many pathways based on various factors, e.g., the level of NADP^+^, GSH, pH, etc. many of the future perspectives in this issue include:

- Detailed studying the interactions between the Pentose Phosphate Pathway (PPP) – as a primary source of NADPH – and MG Pathway, and their possible interrelation with cancer^12^.
- The scientific community should focus in determining the cellular level of MG as a critical determinant of many cellular biochemical pathways (causation) and a powerful tool that tracks the cellular dynamics trajectory (consequences).
- Also, these pathways shed the light on importance of the stereochemistry of the cellular metabolites and their impact on carcinogenesis, besides the stereochemistry of the drugs

These biochemical pathways are involved in carcinogenesis, cancer resistance, and treatment regimes. Therefore, implementing the methylglyoxal pathway in tumour biology represents a promising strategy in the therapeutic approaches against cancer, which can add useful anticancer candidates to the community. These suggested candidates might not be the target of Achilles heels of cancer, but it contributes to rationale of the cancer management.
